# 2-Amino-5-bromo­pyridinium 3-carb­oxy­prop-2-enoate

**DOI:** 10.1107/S1600536810030059

**Published:** 2010-08-04

**Authors:** Madhukar Hemamalini, Hoong-Kun Fun

**Affiliations:** a X-ray Crystallography Unit, School of Physics, Universiti Sains Malaysia, 11800 USM, Penang, Malaysia

## Abstract

In the title salt, C_5_H_6_BrN_2_^+^·C_4_H_3_O_4_^−^, the 2-amino-5-bromo­pyridinium cation and hydrogen maleate anion are planar, with maximum deviations from their mean planes of 0.016 (1) and 0.039 (1) Å, respectively. An intra­molecular O—H⋯O hydrogen bond generates an *S*(7) ring motif in the anion. In the crystal, the protonated N atom and the 2-amino group of the cation are hydrogen-bonded to the carboxyl­ate O atoms of the anion *via* a pair of N—H⋯O hydrogen bonds, forming an *R*_2_^2^(8) ring motif. The motifs are linked into a two-dimensional network parallel to (011) by N—H⋯O and C—H⋯O hydrogen bonds.

## Related literature

For background to the chemistry of substituted pyridines, see: Pozharski *et al.* (1997 ▶); Katritzky *et al.* (1996 ▶). For details of maleic acid, see; Bowes *et al.* (2003 ▶); Jin *et al.* (2003 ▶); Lah & Leban (2003 ▶); Allen (2002 ▶). For bond-length data, see: Allen *et al.* (1987 ▶). For details of hydrogen bonding, see: Jeffrey & Saenger (1991 ▶); Jeffrey (1997 ▶); Scheiner (1997 ▶). For hydrogen-bond motifs, see: Bernstein *et al.* (1995 ▶). For the stability of the temperature controller used in the data collection, see: Cosier & Glazer (1986 ▶).
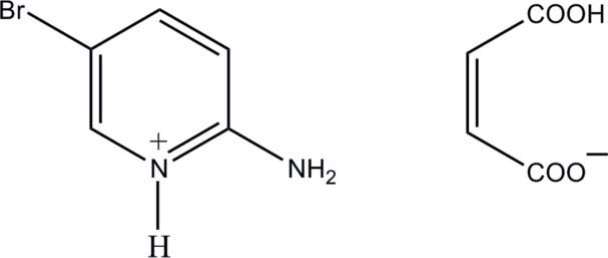


## Experimental

### 

#### Crystal data

C_5_H_6_BrN_2_^+^·C_4_H_3_O_4_^−^
*M* *_r_* = 289.09
Triclinic, 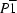

*a* = 5.7434 (1) Å
*b* = 9.5871 (1) Å
*c* = 10.3034 (2) Å
α = 80.455 (1)°
β = 74.175 (1)°
γ = 85.123 (1)°
*V* = 537.80 (2) Å^3^
*Z* = 2
Mo *K*α radiation
μ = 3.82 mm^−1^
*T* = 100 K
0.55 × 0.26 × 0.17 mm

#### Data collection

Bruker SMART APEXII CCD area-detector diffractometer
Absorption correction: multi-scan (*SADABS*; Bruker, 2009 ▶) *T*_min_ = 0.226, *T*_max_ = 0.554
17591 measured reflections
4705 independent reflections
4235 reflections with *I* > 2σ(*I*)
*R*_int_ = 0.020

#### Refinement

*R*[*F* ^2^ > 2σ(*F* ^2^)] = 0.024
*wR*(*F* ^2^) = 0.064
*S* = 1.06
4705 reflections
157 parameters
H atoms treated by a mixture of independent and constrained refinement
Δρ_max_ = 1.11 e Å^−3^
Δρ_min_ = −0.70 e Å^−3^

### 

Data collection: *APEX2* (Bruker, 2009 ▶); cell refinement: *SAINT* (Bruker, 2009 ▶); data reduction: *SAINT*; program(s) used to solve structure: *SHELXTL* (Sheldrick, 2008 ▶); program(s) used to refine structure: *SHELXTL*; molecular graphics: *SHELXTL*; software used to prepare material for publication: *SHELXTL* and *PLATON* (Spek, 2009 ▶).

## Supplementary Material

Crystal structure: contains datablocks global, I. DOI: 10.1107/S1600536810030059/ci5141sup1.cif

Structure factors: contains datablocks I. DOI: 10.1107/S1600536810030059/ci5141Isup2.hkl

Additional supplementary materials:  crystallographic information; 3D view; checkCIF report

## Figures and Tables

**Table 1:** Hydrogen-bond geometry (Å, °)

*D*—H⋯*A*	*D*—H	H⋯*A*	*D*⋯*A*	*D*—H⋯*A*
O1—H1*O*1⋯O3	0.88	1.57	2.4380 (13)	171
N1—H1*N*1⋯O4^i^	0.87 (2)	1.88 (2)	2.7426 (13)	169 (2)
N2—H1*N*2⋯O3^i^	0.84 (2)	2.01 (2)	2.8495 (14)	174 (2)
N2—H2*N*2⋯O2^ii^	0.82 (2)	2.14 (2)	2.9534 (13)	176 (2)
C3—H3*A*⋯O2	0.93	2.37	3.2937 (14)	171
C5—H5*A*⋯O4^iii^	0.93	2.39	3.3051 (14)	167

Symmetry codes: (i) 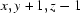; (ii) 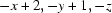; (iii) 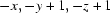.

**Fig. 1.**
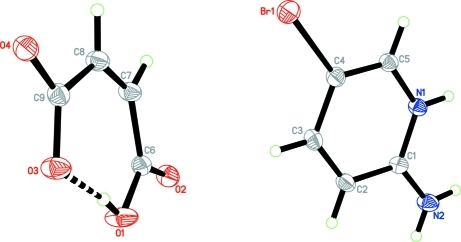
The asymmetric unit of the title compound. Displacement ellipsoids are drawn at the 50% probability level. The dashed line indicates a intramolecular hydrogen bond.

**Fig. 2.**
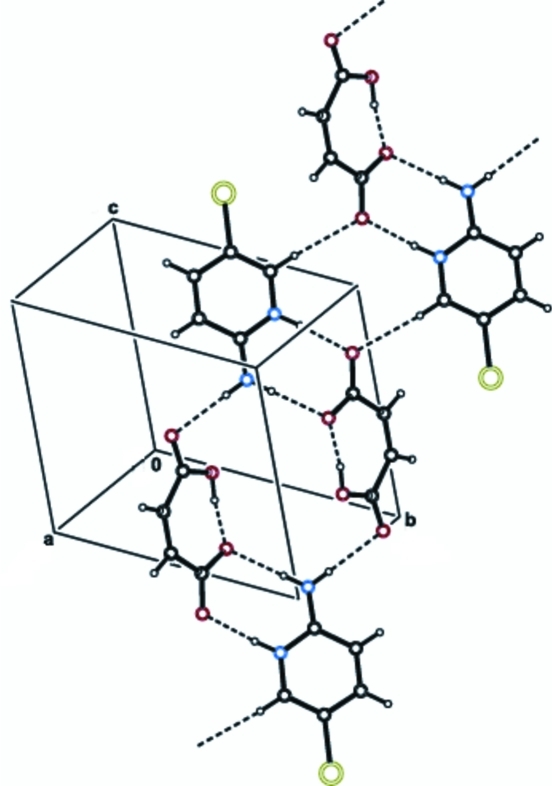
Part of the crystal structure of the title compound, showing S(7) and R22(8) ring motifs.

## supplementary crystallographic information

### Comment

Pyridine and its derivatives play an important role in heterocyclic chemistry (Pozharski *et al.*, 1997; Katritzky *et al.*, 1996). They are often involved in hydrogen-bonding interactions (Jeffrey & Saenger, 1991; Jeffrey, 1997; Scheiner, 1997). Maleic acid, the *Z* isomer of butenedioic acid, has been used as a simple building block in supramolecular architectures in two and three dimensions (Bowes *et al.*, 2003; Jin  *et al.*, 2003). The maleic acid anion can exist in the fully deprotonated form or as hydrogen maleate with one of the carboxyl groups protonated (Lah & Leban, 2003). Several singly dissociated maleate salts are reported in the Cambridge Structural Database (Version 5.29; Allen, 2002). Since our aim is to study some interesting hydrogen-bonding interactions, the crystal structure of the title salt is presented here.

The asymmetric unit (Fig. 1) contains one 2-amino-5-bromopyridinium cation and one hydrogen maleate anion, indicating that proton transfer has occurred during the co-crystallisation experiment. In the 2-amino-5-bromopyridinium cation, a wider than normal angle (C5—N1—C1 = 123.02 (9)°) is subtented at the protonated N1 atom. The 2-amino-5-bromopyridinium cation is essentially planar, with a maximum deviation of 0.016 (1) Å for atom Br1. The bond lengths (Allen *et al.*, 1987) and angles are normal.

In the crystal packing (Fig. 2), the protonated N1 atom and the 2-amino group (N2) are hydrogen-bonded to the carboxylate oxygen atoms (O3 and O4) via a pair of intermolecular N1—H1N1···O4 and N2—H1N2···O3 hydrogen bonds forming an *R*_2_^2^(8) ring motif (Bernstein *et al.*, 1995). There is an intramolecular O1—H1O1···O3 hydrogen bond in the hydrogen maleate anion, which generates an *S*(7) ring motif. Furthermore these two motifs are connected via N2—H2N2···O2, C3—H3A···O2 and C5—H5A···O4 (Table 1) hydrogen bonds, forming a two-dimensional network parallel to the (011) plane.

### Experimental

A hot methanol solution (20 ml) of 2-amino-5-bromopyridine (43 mg, Aldrich) and maleic acid (29 mg, Merck) were mixed and warmed over a heating magnetic stirrer hotplate for a few minutes. The resulting solution was allowed to cool slowly at room temperature and crystals of the title compound appeared after a few days.

### Refinement

Atoms H1N1, H1N2 and H2N2 were located in a difference Fourier map and were refined freely [N–H= 0.82 (2)–0.870 (19) Å ]. The remaining H atoms were positioned geometrically [O–H = 0.88 Å and C–H = 0.93 Å] and were refined using a riding model, with *U*_iso_(H) = 1.2 *U*_eq_(C).

### Crystal data

**Table:**

C_5_H_6_BrN_2_^+^·C_4_H_3_O_4_^−^	*Z* = 2
*M**_r_* = 289.09	*F*(000) = 288
Triclinic, *P*1	*D*_x_ = 1.785 Mg m^−^^3^
Hall symbol: -P 1	Mo *K*α radiation, λ = 0.71073 Å
*a* = 5.7434 (1) Å	Cell parameters from 9953 reflections
*b* = 9.5871 (1) Å	θ = 2.8–35.2°
*c* = 10.3034 (2) Å	µ = 3.82 mm^−^^1^
α = 80.455 (1)°	*T* = 100 K
β = 74.175 (1)°	Plate, colourless
γ = 85.123 (1)°	0.55 × 0.26 × 0.17 mm
*V* = 537.80 (2) Å^3^	

### Data collection

**Table:**

Bruker SMART APEXII CCD area-detector diffractometer	4705 independent reflections
Radiation source: fine-focus sealed tube	4235 reflections with *I* > 2σ(*I*)
graphite	*R*_int_ = 0.020
φ and ω scans	θ_max_ = 35.0°, θ_min_ = 2.1°
Absorption correction: multi-scan (*SADABS*; Bruker, 2009)	*h* = −9→8
*T*_min_ = 0.226, *T*_max_ = 0.554	*k* = −15→15
17591 measured reflections	*l* = −16→15

### Refinement

**Table:**

Refinement on *F*^2^	Primary atom site location: structure-invariant direct methods
Least-squares matrix: full	Secondary atom site location: difference Fourier map
*R*[*F*^2^ > 2σ(*F*^2^)] = 0.024	Hydrogen site location: inferred from neighbouring sites
*wR*(*F*^2^) = 0.064	H atoms treated by a mixture of independent and constrained refinement
*S* = 1.06	*w* = 1/[σ^2^(*F*_o_^2^) + (0.0326*P*)^2^ + 0.177*P*] where *P* = (*F*_o_^2^ + 2*F*_c_^2^)/3
4705 reflections	(Δ/σ)_max_ = 0.001
157 parameters	Δρ_max_ = 1.11 e Å^−^^3^
0 restraints	Δρ_min_ = −0.70 e Å^−^^3^

### Special details

**Table:**

Experimental. The crystal was placed in the cold stream of an Oxford Cryosystems Cobra open-flow nitrogen cryostat (Cosier & Glazer, 1986) operating at 100.0 (1) K.
Geometry. All s.u.'s (except the s.u. in the dihedral angle between two l.s. planes) are estimated using the full covariance matrix. The cell s.u.'s are taken into account individually in the estimation of s.u.'s in distances, angles and torsion angles; correlations between s.u.'s in cell parameters are only used when they are defined by crystal symmetry. An approximate (isotropic) treatment of cell s.u.'s is used for estimating s.u.'s involving l.s. planes.
Refinement. Refinement of F^2^ against ALL reflections. The weighted R-factor wR and goodness of fit S are based on F^2^, conventional R-factors R are based on F, with F set to zero for negative F^2^. The threshold expression of F^2^ > 2σ(F^2^) is used only for calculating R-factors(gt) etc. and is not relevant to the choice of reflections for refinement. R-factors based on F^2^ are statistically about twice as large as those based on F, and R- factors based on ALL data will be even larger.

### Fractional atomic coordinates and isotropic or equivalent isotropic displacement parameters (Å^2^)

**Table:**

	*x*	*y*	*z*	*U*_iso_*/*U*_eq_	
Br1	0.08004 (2)	0.537686 (13)	0.294128 (12)	0.02696 (4)	
N1	0.42643 (16)	0.83409 (10)	−0.01859 (9)	0.01624 (14)	
N2	0.81699 (17)	0.85829 (11)	−0.15773 (10)	0.02185 (17)	
C1	0.65936 (17)	0.78492 (11)	−0.05651 (10)	0.01673 (16)	
C2	0.72501 (19)	0.65511 (12)	0.01629 (11)	0.01905 (18)	
H2A	0.8842	0.6194	−0.0062	0.023*	
C3	0.55552 (19)	0.58268 (11)	0.11897 (11)	0.01939 (18)	
H3A	0.5979	0.4971	0.1659	0.023*	
C4	0.31486 (19)	0.63883 (11)	0.15333 (11)	0.01821 (17)	
C5	0.25417 (18)	0.76411 (11)	0.08418 (10)	0.01714 (16)	
H5A	0.0962	0.8017	0.1069	0.021*	
O1	0.81743 (14)	0.18263 (11)	0.44160 (9)	0.02726 (19)	
H1O1	0.7489	0.1584	0.5288	0.041*	
O2	0.68803 (15)	0.26191 (9)	0.25903 (8)	0.02102 (15)	
O3	0.63826 (14)	0.09096 (10)	0.68047 (9)	0.02256 (16)	
O4	0.27013 (14)	0.06052 (9)	0.82150 (8)	0.01999 (14)	
C6	0.64341 (18)	0.21863 (11)	0.38215 (10)	0.01679 (16)	
C7	0.38534 (18)	0.20548 (12)	0.46279 (11)	0.01807 (17)	
H7A	0.2729	0.2353	0.4128	0.022*	
C8	0.28808 (18)	0.15795 (12)	0.59511 (11)	0.01824 (17)	
H8A	0.1197	0.1616	0.6220	0.022*	
C9	0.40698 (18)	0.09990 (11)	0.70659 (10)	0.01663 (16)	
H1N1	0.381 (3)	0.913 (2)	−0.0621 (19)	0.026 (4)*	
H1N2	0.769 (3)	0.931 (2)	−0.2027 (19)	0.028 (4)*	
H2N2	0.956 (4)	0.827 (2)	−0.183 (2)	0.038 (5)*	

### Atomic displacement parameters (Å^2^)

**Table:**

	*U*^11^	*U*^22^	*U*^33^	*U*^12^	*U*^13^	*U*^23^
Br1	0.02461 (6)	0.02342 (6)	0.02515 (6)	−0.00137 (4)	0.00082 (4)	0.00590 (4)
N1	0.0158 (3)	0.0172 (4)	0.0146 (3)	0.0015 (3)	−0.0040 (3)	−0.0003 (3)
N2	0.0163 (3)	0.0246 (4)	0.0203 (4)	0.0009 (3)	−0.0015 (3)	0.0024 (3)
C1	0.0153 (4)	0.0190 (4)	0.0154 (4)	0.0006 (3)	−0.0039 (3)	−0.0021 (3)
C2	0.0171 (4)	0.0184 (4)	0.0213 (4)	0.0028 (3)	−0.0059 (3)	−0.0023 (3)
C3	0.0209 (4)	0.0162 (4)	0.0206 (4)	0.0022 (3)	−0.0067 (3)	−0.0009 (3)
C4	0.0191 (4)	0.0175 (4)	0.0163 (4)	−0.0004 (3)	−0.0031 (3)	−0.0005 (3)
C5	0.0164 (4)	0.0179 (4)	0.0158 (4)	0.0006 (3)	−0.0031 (3)	−0.0015 (3)
O1	0.0143 (3)	0.0452 (5)	0.0193 (4)	−0.0035 (3)	−0.0053 (3)	0.0062 (3)
O2	0.0205 (3)	0.0239 (4)	0.0163 (3)	0.0014 (3)	−0.0039 (3)	0.0010 (3)
O3	0.0157 (3)	0.0322 (4)	0.0184 (3)	−0.0007 (3)	−0.0058 (3)	0.0021 (3)
O4	0.0193 (3)	0.0209 (4)	0.0162 (3)	0.0023 (3)	−0.0016 (3)	0.0008 (3)
C6	0.0158 (4)	0.0164 (4)	0.0172 (4)	−0.0001 (3)	−0.0041 (3)	−0.0006 (3)
C7	0.0146 (4)	0.0213 (4)	0.0178 (4)	0.0011 (3)	−0.0053 (3)	−0.0006 (3)
C8	0.0146 (4)	0.0211 (4)	0.0177 (4)	0.0009 (3)	−0.0042 (3)	−0.0002 (3)
C9	0.0171 (4)	0.0164 (4)	0.0161 (4)	0.0006 (3)	−0.0046 (3)	−0.0017 (3)

### Geometric parameters (Å, °)

**Table:**

Br1—C4	1.8848 (11)	C4—C5	1.3609 (15)
N1—C1	1.3540 (13)	C5—H5A	0.93
N1—C5	1.3624 (13)	O1—C6	1.3032 (12)
N1—H1N1	0.870 (19)	O1—H1O1	0.88
N2—C1	1.3237 (14)	O2—C6	1.2301 (13)
N2—H1N2	0.842 (19)	O3—C9	1.2796 (12)
N2—H2N2	0.82 (2)	O4—C9	1.2477 (12)
C1—C2	1.4207 (15)	C6—C7	1.4929 (14)
C2—C3	1.3633 (16)	C7—C8	1.3417 (15)
C2—H2A	0.93	C7—H7A	0.93
C3—C4	1.4126 (15)	C8—C9	1.4988 (14)
C3—H3A	0.93	C8—H8A	0.93
			
C1—N1—C5	123.02 (9)	C3—C4—Br1	119.31 (8)
C1—N1—H1N1	119.6 (13)	C4—C5—N1	119.57 (9)
C5—N1—H1N1	117.3 (13)	C4—C5—H5A	120.2
C1—N2—H1N2	119.8 (13)	N1—C5—H5A	120.2
C1—N2—H2N2	120.2 (15)	C6—O1—H1O1	106.9
H1N2—N2—H2N2	120 (2)	O2—C6—O1	120.95 (9)
N2—C1—N1	119.76 (10)	O2—C6—C7	118.87 (9)
N2—C1—C2	122.44 (9)	O1—C6—C7	120.17 (9)
N1—C1—C2	117.79 (9)	C8—C7—C6	130.95 (9)
C3—C2—C1	120.27 (9)	C8—C7—H7A	114.5
C3—C2—H2A	119.9	C6—C7—H7A	114.5
C1—C2—H2A	119.9	C7—C8—C9	130.44 (9)
C2—C3—C4	119.36 (10)	C7—C8—H8A	114.8
C2—C3—H3A	120.3	C9—C8—H8A	114.8
C4—C3—H3A	120.3	O4—C9—O3	123.48 (10)
C5—C4—C3	119.99 (10)	O4—C9—C8	116.75 (9)
C5—C4—Br1	120.70 (8)	O3—C9—C8	119.77 (9)
			
C5—N1—C1—N2	179.73 (10)	Br1—C4—C5—N1	−178.99 (8)
C5—N1—C1—C2	−0.79 (15)	C1—N1—C5—C4	0.00 (16)
N2—C1—C2—C3	−179.37 (11)	O2—C6—C7—C8	177.71 (12)
N1—C1—C2—C3	1.17 (16)	O1—C6—C7—C8	−1.27 (19)
C1—C2—C3—C4	−0.77 (16)	C6—C7—C8—C9	−1.0 (2)
C2—C3—C4—C5	−0.03 (17)	C7—C8—C9—O4	−178.65 (11)
C2—C3—C4—Br1	179.39 (8)	C7—C8—C9—O3	0.33 (18)
C3—C4—C5—N1	0.43 (16)		

### Hydrogen-bond geometry (Å, °)

**Table:**

*D*—H···*A*	*D*—H	H···*A*	*D*···*A*	*D*—H···*A*
O1—H1O1···O3	0.88	1.57	2.4380 (13)	171
N1—H1N1···O4^i^	0.87 (2)	1.88 (2)	2.7426 (13)	169 (2)
N2—H1N2···O3^i^	0.84 (2)	2.01 (2)	2.8495 (14)	174 (2)
N2—H2N2···O2^ii^	0.82 (2)	2.14 (2)	2.9534 (13)	176 (2)
C3—H3A···O2	0.93	2.37	3.2937 (14)	171
C5—H5A···O4^iii^	0.93	2.39	3.3051 (14)	167

Symmetry codes: (i) *x*, *y*+1, *z*−1; (ii) −*x*+2, −*y*+1, −*z*; (iii) −*x*, −*y*+1, −*z*+1.
